# Estimating the budget impact of a Tuberculosis strategic purchasing pilot study in Medan, Indonesia (2018–2019)

**DOI:** 10.1186/s13561-024-00518-2

**Published:** 2024-06-21

**Authors:** Sarah Saragih, Firdaus Hafidz, Aditia Nugroho, Laurel Hatt, Meghan O’Connell, Agnes Caroline, Cheryl Cashin, Syed Imran, Yuli Farianti, Ackhmad Afflazier, Tiara Pakasi, Nurul Badriyah

**Affiliations:** 1 Results for Development, Jakarta, Indonesia; 2 Department of Health Policy and Management, Universitas Gadjah Mada, Yogyakarta, Indonesia; 3 Results for Development, Washington DC, USA; 4 FHI 360, Jakarta, Indonesia; 5 Ministry of Health, Jakarta, Indonesia

**Keywords:** Budget impact, Strategic purchasing, Tuberculosis, National health insurance, Indonesia

## Abstract

**Background:**

Indonesia has the world’s second-highest tuberculosis (TB) burden, with 969,000 annual TB infections. In 2017, Indonesia faced significant challenges in TB care, with 18% of cases missed, 29% of diagnosed cases unreported, and 55.4% of positive results not notified. The government is exploring a new approach called “strategic purchasing” to improve TB detection and treatment rates and offer cost-effective service delivery.

**Objectives:**

We aimed to analyze the financial impact of implementing a TB purchasing pilot in the city of Medan and assess the project’s affordability and value for money.

**Methods:**

We developed a budget impact model to estimate the cost-effectiveness of using strategic purchasing to improve TB reporting and treatment success rates. We used using data from Medan’s budget impact model and the Ministry of Health’s guidelines to predict the total cost and the cost per patient.

**Results:**

The model showed that strategic purchasing would improve TB reporting by 63% and successful treatments by 64%. While this would lead to a rise in total spending on TB care by 60%, the cost per patient would decrease by 3%. This is because more care would be provided in primary healthcare settings, which are more cost-effective than hospitals.

**Conclusions:**

While strategic purchasing may increase overall spending, it could improve TB care in Indonesia by identifying more cases, treating them more effectively, and reducing the cost per patient. This could potentially lead to long-term cost savings and improved health outcomes.

**Supplementary Information:**

The online version contains supplementary material available at 10.1186/s13561-024-00518-2.

## Background

Indonesia has the second-highest tuberculosis (TB) burden in the world, with 969,000 infections per year and a 16% case-fatality ratio [1]. The country also faces a number of health system challenges that stymie efforts to address this high TB burden. It is estimated that 18% of TB cases in Indonesia are missed or undiagnosed, 29% of diagnosed cases are never reported [2], and 55.4% of people who test positive are never notified [2]. Difficulty with containing, treating, and monitoring the TB burden contributes to high mortality, drug resistance, and continued disease transmission.

Indonesians also experience limited financial protection for TB treatment. Although 88.6% of the population is covered by the National Health Insurance (NHI) scheme (*Jaminan Kesehatan Nasional*) [3], a cross-sectional survey of 25 districts found that 38.4% of TB-affected households still experience catastrophic medical costs, where out-of-pocket (OOP) spending on TB care exceeds 20% of household annual income [4].

The NHI currently pays primary health care (PHC) providers to deliver care—including care for uncomplicated TB—using capitation, or a fixed payment per enrollee per month. However, because TB services are costly, PHC providers tend to over-refer presumptive TB cases to hospitals, which are paid via case-based payment. This has contributed to unnecessary expenditures on TB treatment by the Government of Indonesia [5]. It also adds to patients’ time and travel costs since hospitals tend to be farther from patients’ homes, increases initial loss-to-follow-up of confirmed cases, and decreases treatment completion rates.

A strategic health purchasing (SHP) approach that incentivizes PHC providers to deliver TB services could help reduce the TB burden in Indonesia. Such an approach is particularly of interest for engaging private PHC providers in the delivery of better-quality TB care. According to the Joint External TB Monitoring Mission report of 2017, 74% of TB patients initially seek care, and 51% of patients receive treatment from private providers. However, private providers report only 9% of case notifications [6].

Adopting a strategic health purchasing approach means deliberately directing pooled health funds to priority populations and services to achieve the greatest value for money. It involves four core purchasing functions—benefits specification, contracting arrangements, provider payment, and performance monitoring [7]—through which countries can achieve better health outcomes by incentivizing better-quality care and increasing efficiency and equity in spending. In Thailand, strategic purchasing modifications to the health benefits package and payment mechanism reduced catastrophic health spending and increased service utilization [8]. A study conducted in Afghanistan showed similar positive results. It showed that involving the private sector and implementing a pay-for-performance system led to better results. This approach increased the number of people receiving TB care and established a successful system for tracking progress [9].

Despite the limited resources in Indonesia, international bodies, including the Global Fund, greatly support TB control. They are actively working to bridge a significant funding gap, which currently stands at 73% or USD 264 million [1]. Therefore, the Government of Indonesia is investigating the implementation of strategic purchasing within its national TB program (NTP). The aim is to increase the number of TB cases treated and enable more efficient delivery of high-quality, cost-effective care at the PHC level. In this analysis, we propose adding fee-for-service payments for TB diagnostics and episode-based payments for TB treatment. Our model also includes the following: redefining the NHI benefits package to align with the NTP’s clinical guidelines; avoiding double payment and reducing OOP expenditures for TB; modifying NHI credentialing and contracting arrangements to increase TB service provision at the PHC level to improve accessibility for patients; improving the performance monitoring system to link provider payments to the quality of TB services they provide, incentivizing high-quality care.

We created a simulation model using data for Medan City, the capital of North Sumatra, which has a high proportion of private PHC facilities (127 private compared to 52 public facilities) and a high TB burden (832 per 100,000 people). We estimated the cost-effectiveness of implementing these strategic purchasing arrangements, analyzed the financial implications for the NHI and NTP, and discussed the impact on health outcomes and financial protection.

## Methods

We compared the outcomes and costs of two scenarios in Medan: the current (baseline) approach and the proposed SHP approach. We estimated the caseload of presumptive and confirmed TB patients along the clinical care pathway and applied a costing formula for budget impact analysis. We also compared care-seeking and service delivery patterns between public and private facilities.

### Study population

The study focused on individuals with presumptive or confirmed TB from both public and private hospitals, as well as private PHC facilities in the catchment area of 15 public PHC facilities (*puskesmas*) in the city of Medan (see Table 1). These *puskesmas* were selected by the Medan District Health Office for their strong TB reporting and active participation in the District Public–Private Mix network.^1^ The reference population consisted of 1 million individuals enrolled in the NHI program in Medan, representing 95% of the total population across the 15 districts in Medan. Among those enrollees, we projected that 39,778 sought healthcare due to presumptive TB, and out of those, 7,366 were confirmed to have TB. The number of individuals seeking healthcare for TB was obtained using the ratio of 5.4 presumptive TB for every one confirmed TB, derived from Minimum Standard Service monitoring data. Additionally, previous literature, specifically the WHO Inventory study in Indonesia in 2017, estimated that 18% of TB cases in the region went undetected and unreported [2].

**Table 1:** Data and sources for estimating TB cases

Indicator	Number or rate	Source or assumption
Total population of the catchment area of 15 *puskesmas* in Medan	1,136,831	Statistics of Indonesia (2019)
Proportion of the population enrolled in NHI	95%	NHI users in 2019 in Medan
Estimated number of all-type confirmed TB	7,366	18% undetected and unreported—estimate from WHO Inventory study (2018)
Presumptive TB patients seeking care (2018)	39,778	5.4 presumptive TB patients in Medan for each detected TB case—data from the Essential Healthcare Package (2019)

By tracking the journey of TB patients from diagnosis through treatment, we estimated the number of patients who recovered, died, or were lost to follow-up. The proportion of individuals with presumptive TB who sought care from private versus public PHC was derived from sample NHI claims data from 2015 to 2018. We used data from Systematic Intervention for Tuberculosis Treatment (SITT) program to estimate various aspects of TB care among people with presumptive TB, including the types of TB diagnostic tests received, the proportion of people receiving follow-up testing after treatment completion, and the proportion of people who completed or did not complete treatment. We then compared these parameters between public and private healthcare providers. To validate our findings and ensure accuracy, we consulted with experts and key stakeholders within the Ministry of Health (MoH), seeking their consensus.

### Cost analysis

We estimated the current unit costs for outpatient services for drug-sensitive TB (DS-TB) and projected the expected unit costs under the proposed payment methods: fee-for-service for diagnostic services and retrospective episode-based payment for treatment. These estimations were done from the payer’s perspective, taking into account direct medical costs but excluding capitation payments. Unit costs were calculated from the following: 1) sample claims reimbursement data for 2015 to 2018 from *Badan Penyelenggara Jaminan Sosial Kesehatan* (BPJS-K), the country’s NHI agency, 2) logistics pricing from the NTP, 3) 2019 tariffs for visits to private general practitioners from *Badan Pusat Statistik*, the central statistics agency, and 4) 2019 public PHC tariffs set by the government in a district comparable to Medan [1] .

We tallied costs incurred from diagnosis to treatment completion, including treatment monitoring, and identified the respective payers. The payers included NHI, the MoH, donors (such as the Global Fund to Fight AIDS, Tuberculosis and Malaria), and patients themselves (through OOP payments). In the SHP scenario, we considered two types of costs: direct medical costs and the proposed incentive payments to healthcare providers. The direct medical costs include material and labor costs for TB diagnosis and treatment. This includes the costs of diagnostic tests, preventive and anti-TB drugs, diagnostic tests for comorbidities such as HIV and diabetes, and TB-related inpatient and outpatient care. Specifically, inpatient and outpatient care costs include payments for clinical visits, follow-up investigations, medications, and outreach services (see Annex A and B).

The incentive payment scheme was designed to bolster diagnosis and completion rates at the primary care level. We estimated an incentive of IDR 200,000 per patient, which is slightly higher than the estimated IDR 176,000 hospital-level cost of treating uncomplicated TB cases. This higher amount was intentionally set to acknowledge and further motivate primary care providers. For the estimation of diagnostic service costs, we adhered to the current public facility market rates: IDR 7,000 for microscopy and IDR 95,000 for an X-ray.

### Budget impact analysis

We developed a dynamic, open-source spreadsheet tool to estimate the budgetary impact of introducing the strategic purchasing model for pulmonary drug-sensitive TB (DS-TB).^2^ It is based on an algorithm that predicts patient flow over a year, comparing the current approach with the proposed new approach over one year, and incorporates the estimated unit costs of each model. By employing a simple cost-effectiveness analysis, we can estimate the value for money of the proposed model and the costs borne by each payer under that model.

$$\Delta\;Totalcost={Totalcost}_i-{Totalcost}_h$$

h = current situation

i = SHP scenario

$${Total\;\cos t}_{h\;or\;i}=\sum\left(p_{d,t,p}\right)\ast u$$

p = price

p^d^ = price at diagnosis phase

p^t^ = price at treatment phase

p^p^ = price at prevention phase

u= utilization (n)

$$u=N\ast i\ast a\ast s\ast(n_{pr}\;or\;n_{pu})$$

u = utilization (n)

N = total population

i = population incidence (n/100,000)

a = access to health facility (%)

s = presumptive TB (%)

n_pr_ = proportion of people with presumptive TB who can access a private provider (%)

n_pu_ = proportion of people with presumptive TB who can access a public provider (%)

The proportion of patients with presumptive TB is the same in both scenarios because the new approach was not assumed to affect the development of TB symptoms. We used data from relevant studies and made several assumptions to estimate how service delivery patterns would change under the new approach. Our analysis assumes that by implementing the proposed reforms, PHC providers in both the public and private sectors would refer only complicated TB cases to secondary care, thus eliminating unnecessary referrals for diagnostic services. This would result in an 83% decrease in referrals, from 15,914 to 2,770 cases. This referral rate was derived from analysis of BPJS-K sample data, assuming that patients without complications would not be referred.

We assume that under the SHP model, the proportion of confirmed TB cases that are reported to the NTP would increase to 95%. This increase is attributed to the requirement for providers to submit diagnostic testing results in order to receive payment. The overall number of confirmed TB cases reported to the NTP is projected to increase by 64%. This increase in reported cases is assumed to be associated with a rise in the number of patients treated in healthcare facilities. We also assume that under the new scenario, the proportion of cases lost to follow-up at each facility would decrease by 6.47% [10], resulting in an increase in treatment success rate. Hence, the proposed scenario expects that 64% more patients will complete treatment compared to the current scenario. Table 2 summarizes the differences between the current and proposed scenarios.

**Table 2:** Projected service delivery outcomes using the strategic purchasing approach

Parameter	Current scenario (a)	Proposed scenario (b)	%Difference ((b-a)/a)
Number of vertical referrals (from primary to secondary level) for diagnostic services	15,914	2,770	-83%
Number of confirmed TB cases notified (2018)	4,140	6,802	64%
TB patients completing treatment (2018)	3,632	5,939	64%

## Results

The SITT data confirmed that there are variations between public and private PHC providers in terms of diagnostic tests, follow-up tests, and the provision of TB treatment. Table 3 shows that more public providers were serving presumptive TB patients and treating more confirmed TB cases compared to private providers. Data from Medan shows that public PHC providers were more compliant to TB guidelines in treating both presumptive and confirmed TB than private PHC providers. For example, more public providers used GeneXpert diagnostic tests compared to private providers (7% vs. 1%). Additionally, more public providers conducted follow-up tests using microscopy at the end of the second and the fifth months, as well as at the end of treatment. Conversely, private providers preferred to use chest X-rays in diagnosing TB compared to public providers (64% vs. 33%). Despite these differences, the rate of successful TB outcomes did not show a statistically significant difference between private and public providers.

**Table 3:** TB diagnosis and treatment patterns at the PHC facilities in the catchment areas of the 15 puskesmas

Characteristic	Private sector n (%)	Public sector n (%)
Distribution of presumptive TB cases (row % of total reported presumptive TB cases in private and public sector facilities)	2,872 (19%)	12,360 (81%)
Distribution of confirmed TB cases (row % of total confirmed TB cases in private and public sector facilities)	2,219 (44%)	2,792 (56%)
Microscopy tests performed (% of total presumptive TB cases at the facility)	4,897 (171%) ^a^	20,130 (163%) ^a^
GeneXpert tests performed (% of total presumptive TB cases at the facility)	25 (1%)	1,035 (8%)
Chest X-rays performed (% of total presumptive TB cases at the facility)	1,419 (49%)	3,434 (28%)
HIV tests (% of total confirmed TB cases at the facility)	204 (9%)	1,089 (39%)
Glucose tests (% of total confirmed TB cases at the facility)	912 (41%)	1,863 (67%)
Follow-up test at the end of month 2 (% of total cases treated at the facility)	1,194 (54%)	1,930 (80%)
Follow-up test at the end of month 5 (% of total cases treated at the facility)	960 (43%)	1,572 (60%)
Follow-up test at the end of treatment (% of total cases treated at the facility)	1,950 (88%)	2,466 (94%)
Successful treatment (% of cases at the facility)	1,947 (88%)	2,298 (87.58%)
Death (% of cases at the facility)	32 (1.45%)	35 (1.33%)
Patients lost to follow-up (% of cases at the facility)	227 (10.27%)	274 (10.44%)
Failed (% of cases at the facility)	5 (0.23%)	17 (0.65%)

^a^One patient could receive more than one test

The new approach would improve treatment coverage and lead to a short-term increase in total budget outlays due to an increased number of diagnosed TB patients. However, it is estimated to reduce the cost per successfully treated TB patient. The model estimates a 64.3% increase in TB case notifications and a 64.5% rise in successful treatment of notified TB patients under the proposed strategic purchasing approach. In the short term, the payment reforms, including fee-for-service payment for diagnostic tests and episode-based payment for TB treatment, would require a 60% increase in investment for TB diagnosis and treatment. As Table 4 below shows, the total diagnostic costs per presumptive TB patient would increase by 82% as more individuals would receive standardized diagnostic care. However, the new approach is estimated to reduce the cost per treated TB patient by 3% and the cost per successfully treated TB patient by 2%. Furthermore, the treatment cost per patient would fall by 22%.

**Table 4:** Projected total costs and unit costs of intervention in Thousands IDR (USD)

Type of cost	Current (Baseline)	Proposed scenario	Difference
Diagnostic cost per presumptive TB patient	220.2 ($15.49)	400.2 ($28.16)	82%
Treatment cost per treated TB patient	1,475.2 ($103.80)	1,152.1 ($81.06)	-22%
Overall cost per treated TB patient	3,613.9($254.29)	3,515.8 ($247.38)	-3%
Overall cost per successfully treated TB patient	4,118 ($289.83)	4,026 ($283.32)	-2%
Total costs	14,961,208 ($1,052,717)	23,914,171 ($1,682,675)	60%
Cost per capita per month	1.1 ($0,08)	1.7 ($0,12)	60%

1 USD = 14,212 rupiah (Bank Indonesia per 28 Juni 2019)

**Fig. 1:**
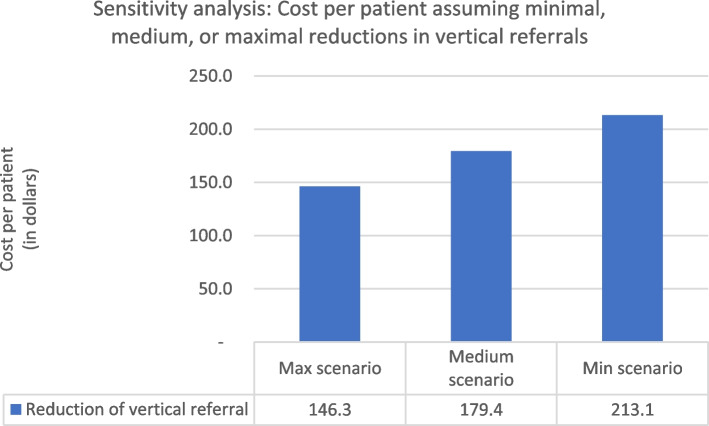
Sensitivity analysis (Cost per patient, varying reductions in vertical referrals) (Minimal scenario: 19% and 8% reduction of upward referrals from private and public PHCs, respectively; Medium scenario: 37% and 15% reduction of upward referrals from private and public PHCs, respectively; Maximal scenario: 56% and 23.1% reduction of upward referrals from private and public PHCs.). *US$1 = 14,212 rupiah (Bank Indonesia, June 28, 2019)

**Fig. 2:**
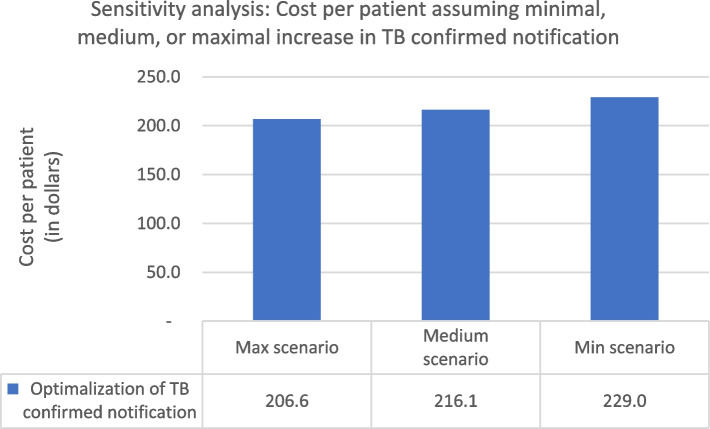
Sensitivity analysis (Cost per patient, varying rates of TB notifications) (Minimal scenario: 30%, 3%, and 19% increase in TB confirmed notification at private PHC, public PHC, and hospitals, respectively; Medium scenario: 60.7%, 6.7%, and 38% increase in TB confirmed notification at private PHC, public PHC, and hospitals; Maximum scenario: 91%, 10%, and 57% increase in TB confirmed notification at private PHC, public PHC, and hospitals.). *US$1 = 14,212 rupiah (Bank Indonesia, June 28, 2019)

To evaluate the robustness of the estimated reduction in overall costs per treated TB patient, we conducted a sensitivity analysis with minimum, medium, and maximum scenarios. We varied three parameters—reduction in upward referrals for TB diagnostics, increased reporting of confirmed TB cases, and increased rates of successful treatment—to see which might have the greatest impact on costs. We found that achieving a larger reduction in vertical referrals to hospitals had the most substantial effect on the cost per patient as shown in Fig. 1. Under the most ambitious assumption of a 56% reduction in upward referrals from private PHCs and a 23% reduction from public PHCs, the overall cost per patient would drop from USD 254.3 to USD 146.3 (42%). Under the assumption of a TB confirmed notification increase minimally across all healthcare facilities, the cost per patient would fall from USD 229.0 to USD 206.6 in the maximum scenario Fig. 2.

Varying the assumptions around rates of TB notifications has a less pronounced effect on the estimated cost per patient.

**Fig. 3:**
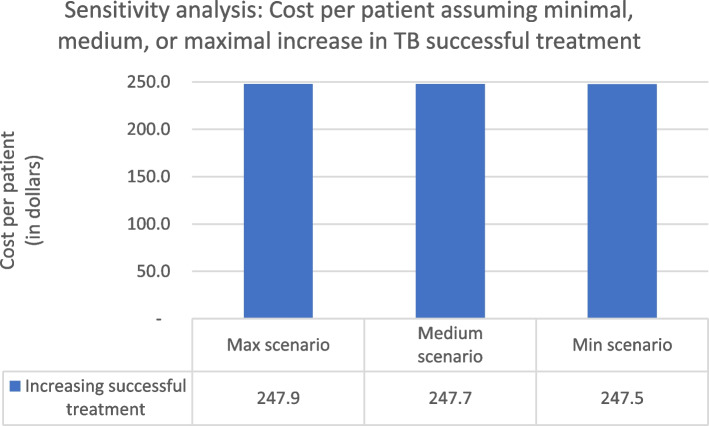
Sensitivity analysis (Cost per patient, varying rates of successful TB treatment) (Minimal scenario: 2.2% increase of successful treatment at private PHC and hospitals, and 1.8% increase of successful treatment at public PHCs; Medium scenario: 4.3% increase of successful treatment at private PHC and hospitals, and 3.5% increase of successful treatment at public PHCs; Maximal scenario: 6.5% increase of successful treatment at private PHC and hospitals, and 5.3% increase of successful treatment at public PHCs.). *US$1 = 14,212 rupiah (Bank Indonesia, June 28, 2019)

However, more optimistic assumptions about the effect of the new approach on rates of successful TB treatment were associated with slightly higher costs per patient (Fig. 3).

We analyzed how the strategic purchasing approach might affect each individual purchaser’s financial outlays. Our analysis showed that the strategic purchasing approach would reduce NHI expenditures but require additional investment from MoH and local government financing schemes (see Table 5). This is because the MoH and local governments are responsible for TB public health activities, supplies for TB diagnostic tests, and TB medications, while external donors, specifically the Global Fund, pay for GeneXpert testing. With more patients being appropriately diagnosed and treated according to national standards, expenditures would accordingly increase. In the first year of implementation, the MoH’s costs for TB diagnosis and treatment would increase by 905% and 68%, respectively. At the same time, the new approach would shift NHI spending from hospitals to primary care facilities. With fewer unnecessary and expensive secondary care referrals, the NHI’s total costs would decrease due to efficiency gains, estimated to be a 23% reduction in the first year. Moreover, OOP payments and related financial burdens on the households would be completely eliminated.

**Table 5:** Estimated costs to the payer for the proposed strategic health purchasing approach (millions of rupiah; U.S. dollars^a^)

Payer	Scenario	Costs of TB diagnostics	Costs of TB treatment	Costs of TB preventive treatment	Total cost
NHI	Current	6,130 ($431,394)	4,281 ($301,251)	-	10,412 ($732,646)
	Proposed	3,172 ($223,242)	4,843 ($340,796)	-	8,016 ($564,039)
	Relative difference	-48%	13%		-23%
Central and local government	Current	1,070 ($75,313)	1,780 ($125,279)	96 ($6,769.20)	2,947 ($207,361)
	Proposed	10,758 ($756,968)	2,993 ($210,602)	158 ($11,122.17)	13,909 ($978,692)
	Relative difference	905%	68%	64%	372%
Global Fund	Current	112 ($7,914)	-	-	112 ($7,914)
	Proposed	1,988 ($139,944)	-	-	1,988 ($139,944)
	Relative difference	1668%			1668%
Out-of-pocket	Current	1,444 ($101,622)	45 ($3,174)	-	1,489 ($104,795)
	Proposed	0	0	0	0
	Relative difference	-100%	-100%		-100%

^a^US$1 = 14,212 rupiah (Bank Indonesia, June 28, 2019)

The increased number of TB cases diagnosed and treatments completed under the new approach would dramatically reduce the number of undetected and untreated TB cases, ultimately slowing the TB epidemic in Indonesia.

## Discussion

The payment mechanisms used by NHI for TB influence how healthcare providers deliver those services. TB is a complex and costly disease to diagnose and treat and has considerable public health implications. Fixed capitation payments, which may reduce risk for payers and have low transaction costs, can demotivate healthcare providers from providing complex or expensive services like TB, leading to increased unnecessary referrals to higher-level facilities [11]. A more tailored purchasing approach could ensure that incentives to provide TB services are strong and aligned with health system objectives. In response to gaps in TB service coverage, the intervention model is adding to the National Health Insurance / *Jaminan Kesehatan Nasional* (JKN) benefit package X-ray examinations, tuberculin tests, and service fees in each test at PHCs. By including these tests, patients could receive comprehensive care without unnecessary referrals, improving access and continuity of treatment. To bolster service quality, the intervention would be integrating TB standards into the JKN credentialing process. This move aimed to align incentives with excellence, particularly for private PHCs, ensuring high-quality care across the healthcare network. Recognizing administrative burdens, the proposed recommendation is to integrate TB reporting and care delivery systems. By linking the TB Information System / *Sistem Informasi Tuberkulosis* (SITB) and P-care (the JKN primary care patient care management system), PHCs could streamline operations and reduce paperwork, allowing them to focus on patient care. In a bid to improve TB service provision, the intervention applies a pay-for-performance mechanism tied to treatment completion. By incentivizing successful outcomes, PHCs were encouraged to prioritize patient adherence and treatment success, driving a culture of excellence in care delivery.

Our model and budget impact analysis (BIA) have yielded the following main conclusions related to TB financing:

- Introducing the SHP approach would increase the number of TB cases notified and successfully treated.
- The cost-effectiveness analysis shows that the new approach would reduce the cost per treated TB patient and the cost per successfully treated TB patient.
- In the current scenario where NTP is responsible for TB diagnostics and medication, the proposed payment modifications would require additional funding from central and local government budgets. However, this would also reduce costs for NHI.

In Medan, more presumptive and confirmed TB cases were recorded in public facilities than in private ones. This contrasts with previous studies showing that patients with presumptive TB often sought care in private facilities [12, 13]. This discrepancy might be due to low rates of TB reporting in private facilities [12]. However, it is important to note that the populations in the studies differ; the earlier study examined the general population nationwide, while our study population was limited to those enrolled in the NHI program.

We found that public PHC facilities are more compliant with TB guidelines compared to private providers. They used GeneXpert tests more frequently for diagnosis and conducted more follow-up tests during and at the end of TB treatment. A study in Yogyakarta also showed that 54.8% of private providers do not comply with NTP guidelines [14]. This difference in adherence to standard TB guidelines could be due to several factors: private providers having limited exposure to International Standards for Tuberculosis Care [14], lack of awareness regarding the importance of microscopy testing [15], objection to several components of the TB guidelines established by NTP, and absence of financial incentives for TB reporting [16].

Our study makes an empirical contribution to knowledge about health financing in general and TB service financing in particular. The proposed strategic purchasing approach considers interactions between payment arrangements at different service levels and types of providers at various healthcare levels. Our findings indicate that combining fee-for-service and episode-based payments for primary-level TB services, along with other purchasing adjustments, could reduce the total cost per treated patient by 3% and the total cost per successful treatment by 2%. These changes in contracting, payment, and monitoring arrangements would incentivize increased TB case finding, notification, and diagnosis and would shift/retain patients to the primary level for treatment. Stronger incentives for monitoring could ensure better treatment adherence, leading to an increased number of patients completing TB treatment successfully.

Our findings are consistent with findings on the positive impacts of SHP in other countries. A study in Taiwan showed that hospitals using pay-for-performance (P4P) incentives in the TB program had lower treatment default rates (10.67%) than hospitals not using P4P (12.7%). Furthermore, P4P hospitals reported a decrease in treatment default rate after implementing the P4P method from 15.56% to 11.37%. [10]. These hospitals also reported a higher number of TB cases cured within nine months of the introduction of the P4P model [17]. P4P approaches have improved provider compliance with guidelines and increased quality of care for chronic diseases [18] and hepatitis B and C [19] in Taiwan and other regions. Additionally, performance-based payment can incentivize higher rates of TB reporting [11]. It should also be noted that the cost per case in our study was marginally lower than the cost per case reported in earlier Indonesia TB studies, which was around USD 242 in 2015 (equivalent to approximately USD 283 in 2019 when adjusted for inflation at an average annual rate of 4%) [20].

Our analysis projects that the TB program will require additional investment from various resources, including the MoH, local governments, and the Global Fund. The main cost drivers are the increased utilization of GeneXpert tests (17 times greater) and TB drugs as more patients are diagnosed and treated. While the Global Fund currently supports GeneXpert testing in Indonesia, this is expected to eventually transition to domestic funding. Conversely, costs borne by the NHI are estimated to decrease by 48% in diagnostic expenses due to decreased referrals from PHC facilities to hospitals and a shift from the higher costs of case-based payments for outpatient TB treatment in hospitals to more cost-effective episode-based payments in PHC facilities.

The shift to PHC will require investments in TB-related clinical and public health functions, including training in contact investigation, counseling, adherence support, and case notification. Similar to South Korea’s success through investment in active contact investigation and comprehensive TB patient management using dedicated TB nurses from both public and private sectors to increase treatment success [21], Indonesia would require substantial investments to increase the number of dedicated TB staff at the PHC level. This would address the expected increase in detected and treated TB patients, with staff focusing on patient coordination, counseling, education and contact investigation, adherence support, and notification. To complement the payment incentives, placing TB case managers in private facilities to ensure that patients receive quality TB services could contribute to a lower treatment and loss to follow-up rate compared to facilities without a case manager [10].

In the immediate term, costs are expected to go up due to increased investments to meet increased demand. However, in the longer term, when investment levels taper off and most new patients have been identified, we project a decrease in TB burden, improved treatment success rates, and reduced treatment costs.

The intervention has been piloted in multiple districts across Indonesia, including Medan. Despite adjustments made during the advocacy process, the model shows promise for implementation in other districts. Success hinges on integrating the model into the existing payment system and ongoing capacity-building efforts.

### Limitations

This research modeled the budget impact of implementing the new approach in Medan only. However, since the model uses standard patient pathways for the treatment of DS-TB according to MoH guidelines, the BIA model parameters could be adapted for use in other areas across Indonesia. The approach can be tailored to show the expected budget impact on a broader scale, such as a wider subset of districts or even a nationwide implementation.

Other limitations of the study include the one-year timeframe and the payers-only perspective, which does not consider indirect patient costs that might affect treatment adherence. We also did not consider costs borne by communities and civil society organizations, which play a significant role in screening and outreach activities. Finally, several of the cost and service delivery parameters were based on assumptions due to a lack of real-world data.

Limitations of available data sources include the following:

- **SITT data (pre-2019):** At the time of data collection, the Tuberculosis (TB) information system utilized was known as SITT (TB Information System). The system has since undergone modifications and is now referred to as SITB (TB Information System) which operates on an online platform and enables real-time reporting. SITT data has numerous limitations. For example, it does not precisely capture the proportion of services used at each type of facility because not all private providers had SITT accounts and therefore some reported manually to *puskesmas*. Additionally, facilities do not always record NHI member numbers, making it difficult to identify all NHI enrollees. However, the SITT database is the only available district-level database that can provide information on presumptive and notified TB patients.
- **BPJS-K sample dataset (2015–2018)**: While this national-level dataset provides valuable insights into NHI service utilization and hospital claims, it is not specifically designed for TB data collection. Therefore, its relevance to TB research may be limited.
- **World Health Organization National Inventory data (2016–2017):** This national-level data, published in 2018, does not capture potential underreporting of TB data at the district level, potentially impacting the accuracy of national-level estimates.

## Conclusions

This study shows that SHP is a cost-effective approach because it reduces the cost per treated TB patient, expands coverage, and reduces OOP spending. In the long run, shifting confirmed uncomplicated DS-TB cases to PHC providers and improving the treatment success rates could reduce the economic burden of TB illness in Indonesia. We believe that our findings could help policymakers choose the appropriate payment mechanisms, promote additional and more effective investments for the TB program to support TB elimination, and provide evidence to start implementing SHP. Future research should adopt a broader societal perspective and consider indirect medical and nonmedical costs borne by civil society and community organizations and organizations to get a more comprehensive picture of TB program financing. Future studies should also consider using real-world data to avoid relying on assumptions in their analytical models.

### Supplementary Information

Supplementary Material 1.

## Data Availability

SITT (2018) is standard reporting system of TB run by Ministry of Health, Department of Infectious disease prevention and control. This is the only available district-level data and can be accessed upon request to District Health Office Medan. The dataset analyzed is already depersonalized and available from the corresponding author on reasonable request. - The BPJS sample dataset 2015-2018 is the sample of nationally representative administrative dataset of BPJS-K in 2015-2018 which launched in 2019. The datasets are available in the BPJSK repository, https://bpjs-kesehatan.go.id/bpjs/ - WHO Global Report 2018 is available in WHO repository, https://www.who.int/teams/global-tuberculosis-programme/tb-reports - WHO Inventory study (2016-2017) report is available publicly (https://cdn.who.int/media/docs/default-source/hq-tuberculosis/global-task-force-on-tb-impact-measurement/meetings/2018-05/tf7_p04_indonesia_inventory_study_results.pdf?sfvrsn=8cd8d1c5_5

## Abbreviations

BIA: Budget impact analysis
BPJS-K: Badan Penyelenggara Jaminan Sosial Kesehatan
DS-TB: Drug-sensitive TB
MoH: Ministry of Health
NHI: National Health Insurance
NTP: National TB Program
OOP: Out-of-pocket
PHC: Primary healthcare
Puskesmas: Public PHC facilities
P4P: Pay-for-performance
SHP: Strategic Health Purchasing
SITT: TB Information system
TB: Tuberculosis
WHO: World Health Organizations

## Footnotes

1 DPPM is a tuberculosis service network within a district that involves all public and private health facilities and is coordinated by the District Health Office (DHO).

2 The model does not account for potential relapse or loss to follow-up among patients who may later develop drug-resistant TB.
