# Supplementary material for: Estimating the budget impact of a Tuberculosis strategic purchasing pilot study in Medan, Indonesia (2018–2019)
Source: Health Econ Rev. 2024 Jun 21;14:44. doi: 10.1186/s13561-024-00518-2 (PMC11191151; doi:10.1186/s13561-024-00518-2)
Supplement: Supplementary file 1 — Supplementary Material 1. [file 13561_2024_518_MOESM1_ESM.docx]

**SUPPLEMENTARY**

ANNEX A. Unit cost per service in current scenario

| **Services** | **Private PHC** | | | | **Public PHC** | | | | **Private Hospital** | | | | **Public Hospital** | | | |
| --- | --- | --- | --- | --- | --- | --- | --- | --- | --- | --- | --- | --- | --- | --- | --- | --- |
|  | **NHI** | **Gov’t budget (national and local)** | **Donors** | **Out-of-pocket** | **NHI** | **Gov’t budget (national and local)** | **Donors** | **Out-of-pocket** | **NHI** | **Gov’t budget (national and local)** | **Donors** | **Out-of-pocket** | **NHI** | **Gov’t budget (national and local)** | **Donors** | **Out-of-pocket** |
| **Diagnosis** |  |  |  |  |  |  |  |  |  |  |  |  |  |  |  |  |
| Microscopy |  |  |  |  |  | 21,444 ($1.5) |  |  | 424,610 ($29.8)*** |  |  |  | 353,842 ($24.9)*** | 21,444 ($1.5) |  |  |
| X-ray |  |  |  | 95,000 ($6.7) |  |  |  | 95,000 ($6.7) |  |  |  |  |  |  |  |  |
| Tuberculin test |  |  |  | 125.000 |  | 78,125 ($5.5) |  |  |  |  |  |  |  | 78,125 ($5.5) |  |  |
| GenXpert molecular testing |  | 295,000 ($20.8) | 50,000 ($3.5) |  |  | 295,000 ($20.8) | 50,000 ($3.5) |  |  | 295,000 ($20.8) | 50,000 ($3.5) |  |  | 295,000 ($20.8) | 50,000 ($3.5) |  |
| **Prevention** |  |  |  |  |  |  |  |  |  |  |  |  |  |  |  |  |
| Isoniazid prophylaxis for children |  | 18,749 ($1.3) |  |  |  | 18,749 ($1.3) |  |  |  | 18,749 ($1.3) |  |  |  | 18,749 ($1.3) |  |  |
| Isoniazid for people with HIV |  | 213,637 ($15) |  |  |  | 213,637 ($15) |  |  |  | 213,637 ($15) |  |  |  | 213,637 ($15) |  |  |
| **Inpatient treatment** | 220,000 ($15.5) |  |  |  | 220,000 ($15.5) |  |  |  |  | 7,247,885 ($510) |  |  | 6,039,904 ($425) |  |  |  |
| **Outpatient treatment** |  |  |  |  |  |  |  |  |  |  |  |  |  |  |  |  |
| HIV test |  |  |  | 48,000 ($3.38) |  | 48,000 ($3.38) |  |  | 212,305 ($14.9)**** |  |  | 48,000 ($3.38) | 176,921 ($12.5)**** | 48,000 ($3.38) |  |  |
| Diabetes test |  |  |  | 20,000 ($1.4) |  |  |  | 20,000 ($1.4) |  |  |  |  |  |  |  |  |
| Clinical visit | - |  |  |  | - |  |  |  | 1,698,442 ($119.5) |  |  |  | 1,415,368 ($99.6) |  |  |  |
| Follow-up bacteriology test |  |  |  |  |  |  |  |  |  |  |  |  |  |  |  |  |
| Initiation phase | - |  |  |  |  | 10,722 ($0.75) |  |  |  |  |  |  |  | 10,722 ($0.75) |  |  |
| Continuation phase | - |  |  |  |  | 10,722 ($0.75) |  |  |  |  |  |  |  | 10,722 ($0.75) |  |  |
| End of treatment | - |  |  |  |  | 10,722 ($0.75) |  |  |  |  |  |  |  | 10,722 ($0.75) |  |  |
| Medication |  | 364,652 ($25.6) |  |  |  | 364,652 ($25.6) |  |  |  | 364,652 ($25.6) |  |  |  | 364,652 ($25.6) |  |  |
| Patient Outreach | - |  |  |  |  | 150,000 ($10.5) |  |  |  | 150,000 ($10.5) |  |  |  |  |  |  |

*US$1 = 14,212 rupiah (Bank Indonesia, June 28, 2019)

ANNEX B. Unit cost per service in SHP scenario

| **Services** | **Private PHC** | | | | **Public PHC** | | | | **Private Hospital** | | | | **Public Hospital** | | | |
| --- | --- | --- | --- | --- | --- | --- | --- | --- | --- | --- | --- | --- | --- | --- | --- | --- |
|  | **NHI** | **Gov’t budget (national and local)** | **Donors** | **Out-of-pocket** | **NHI** | **Gov’t budget (national and local)** | **Donors** | **Out-of-pocket** | **NHI** | **Gov’t budget (national and local)** | **Donors** | **Out-of-pocket** | **NHI** | **Gov’t budget (national and local)** | **Donors** | **Out-of-pocket** |
| **Diagnosis** |  |  |  |  |  |  |  |  |  |  |  |  |  |  |  |  |
| Microscopy | 7,000 ($0.5) | 21,444 ($1.5) |  |  | 7,000 ($0.5) | 21,444 ($1.5) |  |  | 424,610 ($29.8)*** |  |  |  | 353,842 ($24.9)*** | 21,444 ($1.5) |  |  |
| X-ray | 95,000 ($6.7) |  |  |  | 95,000 ($6.7) |  |  |  |  |  |  |  |  |  |  |  |
| Tuberculin test | 46,875 ($3.3) | 78,125 ($5.5) |  |  | 46,875 ($3.3) | 78,125 ($5.5) |  |  |  |  |  |  |  | 78,125 ($5.5) |  |  |
| GenXpert molecular testing |  | 295,000 ($20.8) | 50,000 ($3.5) |  |  | 295,000 ($20.8) | 50,000 ($3.5) |  |  |  | 295,000 ($20.8) | 50,000 ($3.5) |  | 95,000 ($20.8) | 50,000 ($3.5) |  |
| **Prevention** |  |  |  |  |  |  |  |  |  |  |  |  |  |  |  |  |
| Isoniazid prophylaxis for children |  | 18,749 ($1.3) |  |  |  | 18,749 ($1.3) |  |  |  | 18,749 ($1.3) |  |  |  | 18,749 ($1.3) |  |  |
| Isoniazid for people with HIV |  | 213,637 ($15) |  |  |  | 213,637 ($15) |  |  |  | 213,637 ($15) |  |  |  | 213,637 ($15) |  |  |
| **Inpatient treatment** | 220,000 ($15.5) |  |  |  | 220,000 ($15.5) |  |  |  | 7,247,885 ($510) |  |  |  | 6,039,904 ($425) |  |  |  |
| **Outpatient treatment** |  |  |  |  |  |  |  |  |  |  |  |  |  |  |  |  |
| Medications |  | 364,652 ($25.6) |  |  |  | 364,652 ($25.6) |  |  |  | 364,652 ($25.6) |  |  |  | 364,652 ($25.6) |  |  |
| ***First treatment phase*** |  |  |  |  |  |  |  |  |  |  |  |  |  |  |  |  |
| HIV test | 7,000 ($0.5) | 48,000 ($3.38) |  |  | 7,000 ($0.5) | 48,000 ($3.38) |  |  | 212,305 ($14.9) |  |  |  | 176,921 ($12.5) | 48,000 ($3.38) |  |  |
| Diabetes test | 20,000 ($1.4) |  |  |  | 20,000 ($1.4) |  |  |  |  |  |  |  |  |  |  |  |
| Clinical visit | 186,668 ($13.1) |  |  |  | 186,668 ($13.1) |  |  |  |  |  |  |  |  |  |  |  |
| Follow-up bacteriology test (1) | 7,000 ($0.5) | 10,722 ($0.75) |  |  | 7,000 ($0.5) | 10,722 ($0.75) |  |  | 1,698,442***** ($119.5) |  |  |  | 1,415,368***** ($99.6) |  |  |  |
| Patient outreach |  | 150,000 ($10.5) |  |  |  | 150,000 ($10.5) |  |  |  | - |  |  |  |  |  |  |
| ***Second treatment phase*** |  |  |  |  |  |  |  |  |  |  |  |  |  |  |  |  |
| Follow-up bacteriology test (2) | 14,000 ($0.99) | 21,444 ($1.5) |  |  | 14,000 ($0.99) | 21,444 ($1.5) |  |  |  |  |  |  |  |  |  |  |
| Clinical visit | 186,668 ($13.1) |  |  |  | 186,668 ($13.1) |  |  |  |  |  |  |  |  |  |  |  |
| Patient outreach |  | 150,000 ($10.5) |  |  |  | 150,000 ($10.5) |  |  |  | - |  |  |  | - |  |  |
| **Incentive for treatment completion** | 200,000 ($14.1) |  |  |  | 200,000 ($14.1) |  |  |  | - |  |  |  | - |  |  |  |

*US$1 = 14,212 rupiah (Bank Indonesia, June 28, 2019)

** Unit cost per visit at public hospital: 179,921; unit cost per visit at private hospital: 212,305 (20% higher than public hospital)

***Estimation, two times visits at hospital level

****Estimation, one visit at hospital level

***** Estimation, eight visits at hospital level
